# Estimating Heart Rate, Energy Expenditure, and Physical Performance With a Wrist Photoplethysmographic Device During Running

**DOI:** 10.2196/mhealth.7437

**Published:** 2017-07-25

**Authors:** Jakub Parak, Maria Uuskoski, Jan Machek, Ilkka Korhonen

**Affiliations:** ^1^ BioMediTech Institute Faculty of Biomedical Sciences and Engineering Tampere University of Technology Tampere Finland; ^2^ PulseOn Oy Espoo Finland; ^3^ Department of Biology of Physical Activity University of Jyväskylä Jyväskylä Finland

**Keywords:** fitness trackers, photoplethysmography, heart rate, heart rate determination, exercise test, oxygen consumption, energy metabolism

## Abstract

**Background:**

Wearable sensors enable long-term monitoring of health and wellbeing indicators. An objective evaluation of sensors’ accuracy is important, especially for their use in health care.

**Objective:**

The aim of this study was to use a wrist-worn optical heart rate (OHR) device to estimate heart rate (HR), energy expenditure (EE), and maximal oxygen intake capacity (VO_2Max_) during running and to evaluate the accuracy of the estimated parameters (HR, EE, and VO_2Max_) against golden reference methods.

**Methods:**

A total of 24 healthy volunteers, of whom 11 were female, with a mean age of 36.2 years (SD 8.2 years) participated in a submaximal self-paced outdoor running test and maximal voluntary exercise test in a sports laboratory. OHR was monitored with a PulseOn wrist-worn photoplethysmographic device and the running speed with a phone GPS sensor. A physiological model based on HR, running speed, and personal characteristics (age, gender, weight, and height) was used to estimate EE during the maximal voluntary exercise test and VO_2Max_ during the submaximal outdoor running test. ECG-based HR and respiratory gas analysis based estimates were used as golden references.

**Results:**

OHR was able to measure HR during running with a 1.9% mean absolute percentage error (MAPE). VO_2Max_ estimated during the submaximal outdoor running test was closely similar to the sports laboratory estimate (MAPE 5.2%). The energy expenditure estimate (n=23) was quite accurate when HR was above the aerobic threshold (MAPE 6.7%), but MAPE increased to 16.5% during a lighter intensity of exercise.

**Conclusions:**

The results suggest that wrist-worn OHR may accurately estimate HR during running up to maximal HR. When combined with physiological modeling, wrist-worn OHR may be used for an estimation of EE, especially during higher intensity running, and VO_2Max,_ even during submaximal self-paced outdoor recreational running.

## Introduction

Advances in wearable sensors enable long-term monitoring of health and wellbeing indicators in various conditions and activities in both consumers and patients. Recently, significant progress in the size, power consumption, and accuracy of various different sensing technologies has led to an introduction of affordable wearable sensors with a reasonable battery life and capability to monitor, for example, physical activity, sleep, heart function, and so on. However, the reliability and accuracy of the produced information has been questioned and significant differences between different brands have been found [1]. Therefore, an objective scientific evaluation of available wearable sensors is essential for the progress of their use, especially for health applications such as chronic disease prevention and management.

Heart rate (HR) monitoring provides valuable information on physiology and health status during sports, daily life, and sleep. Chest strap HR monitors have been used during sports to quantify and control training loads since the late 1980s. The main limitation for the wide and long-term use of chest strap HR monitors, especially in female users, is the discomfort that is caused by the tightness of the chest strap and possible skin irritations. Therefore, their application has remained relatively limited, especially in real-life wearable monitoring.

Wearable optical HR (OHR) monitoring technology based on photoplethysmography (PPG) has been significantly improved recently because of miniaturized low-power hardware and improved embedded algorithms. OHR technology can be applied on almost any part of the body, such as on the wrist, and can hence overcome some challenges of chest strap HR monitors in their usability and long-term use. However, relatively few scientific studies have reported OHR technology performance and accuracy in laboratory or real-life conditions. Olenick et al evaluated a Mio Alpha wrist OHR device during a graded treadmill exercise test until volitional fatigue and found a strong correlation between OHR and ECG-based HR [2]. In a study by Parak and Korhonen [3], wrist and forearm OHR devices were evaluated during multiple physical activities (walking, running, and biking) with a 5% agreement ranging from 76% to 78%. Delgado-Gonzalo et al evaluated the accuracy and reliability of two different wrist OHR devices (PulseOn and Mio Alpha) against ECG-derived HR in laboratory conditions during a wide range of physical activities and found the mean absolute error of PulseOn to be 3% and Mio Alpha to be 6% during laboratory protocol [4]. Similar or better accuracy was seen during normal outdoor sports activities [4]. In general, wrist-worn OHR devices seem to provide good accuracy during running, but less so in some other activities, such as biking and weight lifting [5-7]. These studies suggest that the currently available high-end OHR devices are reaching acceptable accuracy for HR monitoring during cardiovascular sports such as running, while different brands and devices may experience significant differences in their performance.

Exercise HR is itself a valuable parameter. For example, it allows a real time control of training loads. However, exercise HR alone is challenging to interpret for users, and an estimation of more advanced physiological parameters during exercise would be beneficial to allow a more insightful analysis of the training. An estimation of momentary oxygen consumption and total energy expenditure (EE) for each training session and an estimation of changes in physical performance achieved by regular training are examples of these insightful parameters. An indirect calorimeter is one of the most accurate reference methods for estimating EE. This method is based on the analysis of respiratory gases and is commonly used in laboratory settings. HR has also been used for estimating oxygen consumption. Montgomery et al [8] evaluated the accuracy of oxygen consumption and EE estimation based on chest strap HR monitors and found a slight underestimation with a 6% coefficient of a variation of 6% for oxygen consumption and 13% for EE. Keytel et al [9] reported a correlation coefficient of .913 between the chest strap HR-based method and indirect calorimeter-based EE. Running speed can also be used to estimate oxygen consumption; in runners, a strong correlation (>.99) has been reported [10,11]. Robertson et al [12] found a significant correlation between EE estimates based on indirect calorimetry and a HR chest strap based method during low intensity exercise and maximum intensity exercise. However, Wallen et al observed poor accuracy in an EE estimation of four OHR smart watches as compared with indirect calorimetry [13].

Physical performance may be estimated by the maximal oxygen consumption (VO_2Max_) of a person. VO_2Max_ can be measured directly with an expiratory gas analyzer during a maximal voluntary exercise test. Running speed may also be used to estimate VO_2Max_ [14]. By estimating the oxygen consumption and speed during submaximal exercise, it is possible to estimate VO_2Max_ without maximal exercise testing [15]. LeBoeuf et al found good accuracy of an OHR sensor placed in the ear in the assessment of EE and VO_2Max_: −0.7 (SD 7.4%) and −3.2 (SD 7.3%) [16]. However, to our knowledge, the accuracy of a wrist-worn OHR on the estimation of EE, oxygen consumption, or VO_2Max_ has not been widely studied.

The objectives of the current study were to use OHR to estimate HR, EE, and VO_2Max_ during running and to evaluate the accuracy of the estimated parameters (HR, EE, and VO_2Max_) against a chest strap HR and respiratory gas analysis derived from golden reference values.

## Methods

### Subjects

Twenty-four healthy adults (13 males and 11 females) participated in the study (Table 1). The inclusion criteria were age (18-55 years), BMI (18-30), normal self-reported health status, experience in treadmill running, and a self-estimated ability and willingness to continue the exercise protocol with an increasing load until exhaustion. The health status of the subjects was evaluated in advance through a self-reporting questionnaire and a verbal interview by a trained sports laboratory physiologist about the subjects’ capabilities to reach maximum performance. The subjects provided signed informed consent to participate in the study and they were told that they could withdraw from the study or protocol at any time, if they so desired. The study followed the ethical guidelines of the Helsinki declaration.

**Table 1:** Demographics of the participants.

Parameter	All	Male	Female
No. of participants	24	13	11
Age in years, mean (SD)	36.2 (8.2)	36.8 (9.1)	35.4 (7.2)
Height in cm, mean (SD)	174.1 (8.0)	180.0 (5.6)	167.2 (3.5)
Weight in kg, mean (SD)	69.2 (10.6)	76.1 (9.0)	61.1 (5.2)
BMI^a^ in kg/m^2^, mean (SD)	22.7 (1.9)	23.4 (1.8)	21.8 (1.7)

^a^BMI: body mass index.

### Study Protocol

The study protocol included two parts: (1) a submaximal outdoor running test and (2) a maximal voluntary exercise test in the sports laboratory. The submaximal outdoor running test was performed in regular outdoor conditions in Finland with the aim of providing data from uncontrolled and sometimes challenging conditions, where subjects would train and perform their fitness tests when provided with self-testing equipment, such as a PPG wrist device and a mobile phone. The data from the submaximal outdoor running tests was used to estimate VO_2Max,_ based on wrist PPG and mobile phone GPS data. The maximal voluntary exercise was performed to provide a standardized reference (“ground truth”) for VO_2Max_ for each individual and to compare EE from a wrist PPG against a standard respiratory gas analysis-based EE reference during running. The order of the tests was randomized with a maximal time difference of 7 days.

The submaximal outdoor running test was performed on a pre-defined outdoor track with a flat surface. The subjects were instructed to run at a self-determined pace for at least 20 min, targeting moderate to vigorous subjectively assessed intensity, and to run 5 km. HR was monitored with an optical wrist worn heart rate monitor (PulseOn, Espoo, Finland) and GPS data with a mobile phone (Samsung S3 Galaxy Trend). A Polar V800 HR monitor (Polar Electro, Kempele, Finland) with a built-in GPS sensor was used as a reference for the distance. The GPS reference for the distance was necessary, as the subjects performed the actual running test without continuous supervision and, hence, had a possibility to vary their running route to some extent. The PulseOn mobile app was used to track and store HR and running speed during the test. Field tests were performed outdoors between November 2014 and January 2015 in Finland in regular winter training conditions, that is, during days when it was not raining or snowing, the testing track was not too slippery to cause health risks, and the temperature was above -10 °C. The subjects were instructed to wear their own outdoor sports clothing as appropriate for the current weather during the test. These conditions are typical outdoor training conditions in Finland and, hence, provide a good benchmark for challenging real outdoor training conditions that are faced by ordinary citizens while training.

The maximal voluntary exercise test was performed in a sports testing laboratory with a treadmill (OJK-2, Telineyhtymä, Kotka, Finland). The indoor temperature during the tests was 20 °C. During the test, the subjects wore a face mask from the respiratory gas analyzer (Metalyzer 3B, Metasoft Studio 4.8, Cortex Biophysik GmbH, Leipzig, Germany), the PulseOn wrist HR device, and a chest strap HR device (RS800CX, Polar Electro, Kempele, Finland). The treadmill inclination was set to 0.6°. After setting up the measurement devices and instructing the user about the study protocol and the use of the treadmill, the subject performed a warm-up run at 8 km/h for 6 min. Then, the subject stood still for 6 min and the first blood sample was taken, after which the actual test started. The running speed was increased by 1 km/h, which was maintained for 3 min to reach a stable metabolism at each load. The initial running speed was set so that the predicted number of loads that the subject would be able to complete would be between 8 and 10. Between transitions, the treadmill was stopped for 20-30 s, during which a blood sample was drawn from the subject’s finger to estimate the blood lactate (Biosen C_Line, EKF Diagnostic, 42 Barleben/Magdeburg, Germany). The test was continued until the subject wanted to stop (a stop signal was agreed upon in advance) or the following end criteria, based on recommendations by the Finnish Sports Testing Society, were met: (1) predicted maximum heart rate was reached, (2) measured VO_2_ was stabilized or started to decrease, (3) blood lactate level increased above a threshold, or (4) respiratory exchange ratio was >1.1. After the test, the subject was allowed to recover for 3 min, which was followed by a 7 min cool down jog at a self-selected speed. After this, the final blood sample was taken.

### Energy Expenditure and Maximal Oxygen Intake Capacity Estimation From Optical Heart Rate

PulseOn OHRs recorded during submaximal and maximal tests were re-analyzed offline because of the randomized order of the field and laboratory tests. VO_2Max_ was calculated from the submaximal test and EE was calculated from the maximal exercise test. HR, GPS data, and personal subject information (height, weight, gender, and age) were used for calculations. Both maximal HR estimated during the maximal exercise test and maximal HR estimated from the subject’s age (208 − 0.7 × age [17]) were used for the VO_2Max_ calculation. VO_2Max_ estimated offline from the submaximal test was used for the EE estimation during the maximal exercise test.

The estimation of total EE was based on a method developed earlier [18]. Neural networks were used to derive momentary oxygen consumption (VO_2_) from HR. Differences in the HR-VO_2_ relationship during the different exercise phases (on and off phases) were included in the model. Personal maximal HR and estimated VO_2Max_ were used for the calculation of the momentary VO_2_ value. EE was then estimated from VO_2_, respiratory quotient (RQ), and caloric equivalent [18]. RQ describes the ratio between carbon dioxide produced and oxygen consumed in metabolism, varying from 0.70 to 1.00. RQ has a well-established deterministic relationship with the caloric equivalent, which describes the amount of energy expended per one liter of consumed oxygen, varying from 4.69 to 5.05 kcal/l O_2_ [19]. Both exercise intensity and duration affect the RQ and caloric equivalent. An increase in exercise intensity results in an increased RQ and caloric equivalent, due to the increased oxidation of carbohydrate and decreased oxidation of fat. A prolonged exercise duration has an opposite effect, due to the increased oxidation of fat and decreased oxidation of carbohydrate. When the momentary VO_2_ and caloric equivalent are known, it is possible to calculate the momentary EE. The total EE can be calculated by summing up the momentary EE values.

VO_2Max_ was estimated from OHR and GPS speed recorded during the self-paced running test by a company (Firstbeat, Jyväskylä, Finland) [20]. The method is based on a linear relationship between VO_2_ and the running speed. First, speed and OHR data are segmented to different HR ranges and the reliability of different data segments is estimated by calculating the correlation between HR and speed and comparing that to the variance of the data in that segment. In case of a wide variance and low correlation, the segment is discarded as being unreliable. Then, the most reliable data segments are used to estimate VO_2Max_ by utilizing the relationship between HR and speed. Finally, VO_2Max_ is estimated as the reliability weighted average of the segments.

### Data Analysis

A maximal voluntary exercise test was used to determine the reference (“ground truth”) VO_2Max,_ as well as measure EE during the test. EE was measured by averaging the measured EE, based on a respiratory gas analysis for each minute. Equations defined by Weir [21] were used to calculate EE, based on respiratory gas measurements. VO_2Max_ was determined by using criteria defined by the Finnish Society of Sport Sciences [22].

HR data from a chest belt acquired during the laboratory test was analyzed with Firstbeat Sports software (Firstbeat, Jyväskylä, Finland, version 4.5). After applying an artifact correction algorithm to the signals, the maximum HR value was observed. A second-by-second chest strap HR was used as a reference for the OHR signal during the maximal voluntary test, and the acquired maximum HR value was used as the measured maximum HR in the further analysis.

### Statistical Analyses

The HR estimation accuracy of the wrist PPG device was estimated during the maximum exercise test by comparing HR from the wrist PPG device with chest strap-based HR. First, the data were re-sampled at 1.5 s sampling intervals. HR signals were synchronized in time by maximizing the cross-correlation between the signals at *t*=0. Then, the HR data was averaged over 5 s non-overlapping windows. HR accuracy was estimated by the following parameters [3,4].

Reliability: The percentage of time that the absolute error is smaller than 10 bpm.

Accuracy: The complement of the relative error (ie, 100% mean absolute percentage error).

The difference between VO_2Max_ estimated with a wrist PPG device and GPS data during a submaximal test and with a gas analyzer during a maximal exercise test was compared by calculating the bias, mean absolute error (MAE), mean absolute percentage error (MAPE), and correlation coefficient (either Pearson when data was normally distributed or Spearman when this was not the case) between the estimates. Bland-Altman plots were constructed to allow a visual presentation of the agreement between the two estimation methods and their average error (bias), as well as 95% confidence limits of agreement.

The difference between EE estimated from the wrist PPG device and respiratory gas analysis was calculated during the maximum exercise test. The analysis was carried out separately for light intensity (below aerobic threshold) and medium heavy intensity (between aerobic and anaerobic thresholds). The estimation was only performed from light to medium heavy intensity levels, as higher intensity levels can change the body acid-base balance, which can distort the indirect calorimetry method [23]. The aerobic and anaerobic thresholds of the subjects were determined by the guidelines of the Finnish Society of Sport Sciences [22,24]. Bland-Altman plots were generated for a visual analysis of the error, and bias, MAE, MAPE, and correlation coefficients were calculated for the data.

The normal distribution of data was examined by the Shapiro-Wilk test. The difference between the methods was tested with a paired *t* test in case normal distribution was confirmed and with the Wilcoxon signed rank test when normal distribution could not be confirmed. Pearson correlation coefficient was computed between normally distributed parameters, while Spearman rank correlation coefficient was used for the other parameters not meeting the normal distribution assumption. The strength of the correlation coefficients was interpreted based on the following definitions: weak (*r* ≤.5), moderate (*r*=.5–.7) and strong (*r* ≥.7). All statistical tests were performed as two-sided and the level of significance was set at *P*<.05.

All data analysis was carried out with MathWorks Matlab (version 8.5). All statistical testing was carried out with IBM SPSS statistics (version 22).

## Results

### Heart Rate Accuracy During Treadmill Running

**Figure 1:**
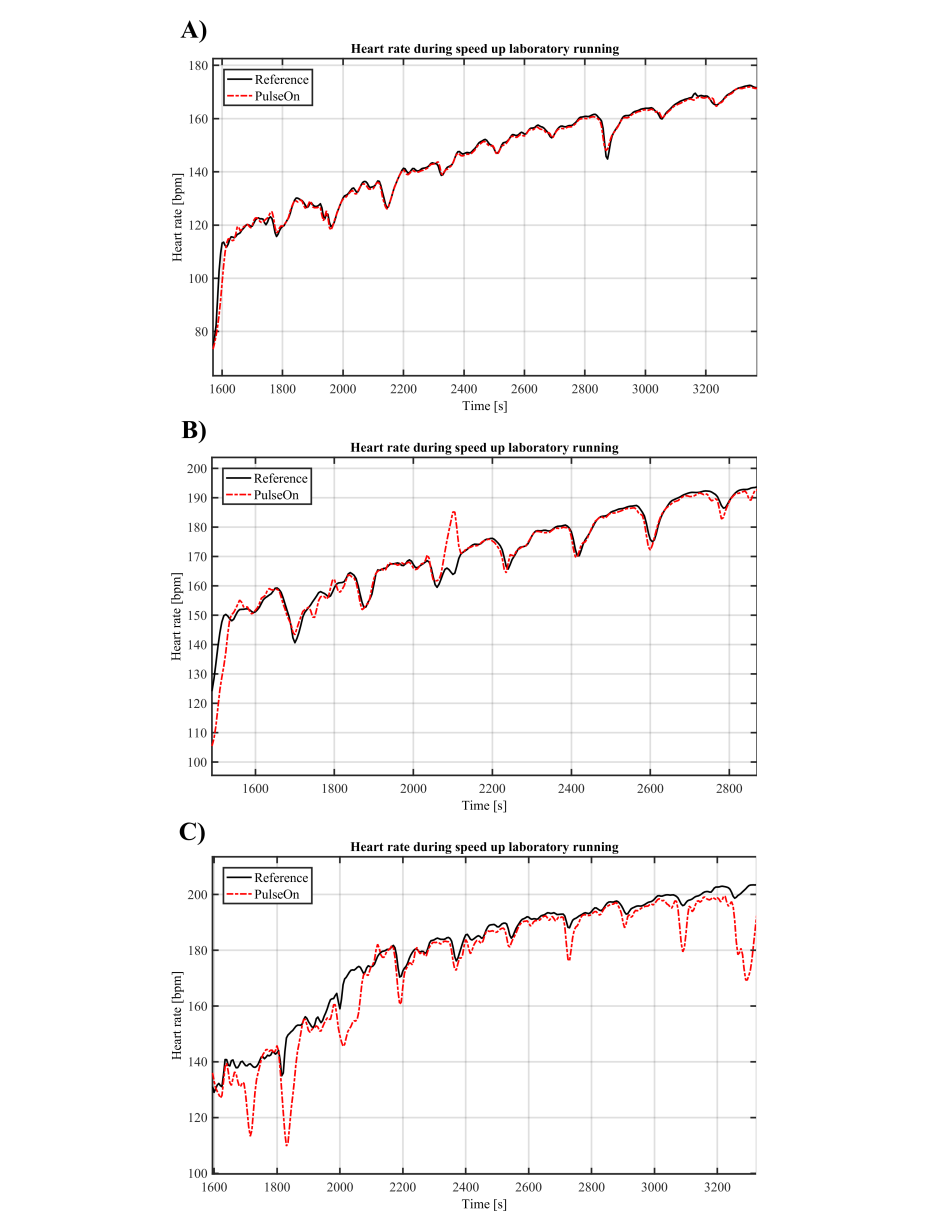
Comparison of HR from chest strap (black line) and wrist PPG device (red line) during maximum exercise test: (A) best accuracy, (B) average accuracy, and (C) worst case.

**Figure 2:**
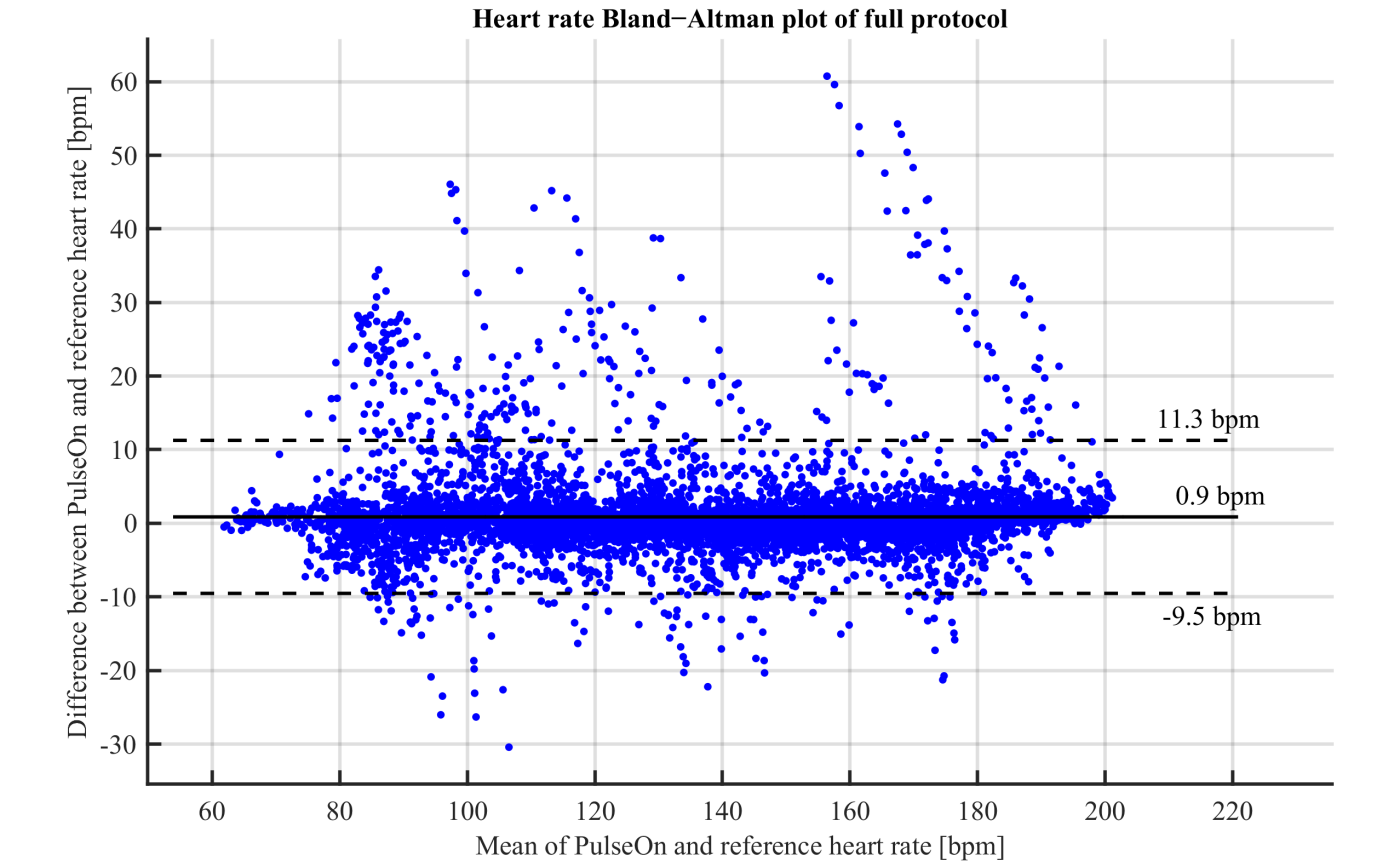
Bland−Altman plot comparing the wrist PPG device and chest strap HR device during maximum exercise protocol in all 24 subjects (solid horizontal line: bias, dashed lines: 95% confidence limits of agreement).

HR estimated with a wrist PPG device appeared to closely follow HR monitored with a chest strap (Table 2). In most cases, wrist PPG HR estimated HR accurately over the entire protocol, even up to maximum HR and running speeds, as shown in Figure 1 (parts A and B). In a few cases, there were occasional outliers, as shown in Figure 1 (part C: in the worst case, OHR artifacts during the beginning of the recording are likely related to poor perfusion before fully warming up, while at the end, the subject was struggling to maintain the running speed, resulting in non-rhythmic hand motions because the subject was aiming to gain support from the treadmill handles.). This can also be seen in Figure 2, which presents the Bland-Altman plot of the HR during the entire laboratory protocol from a wrist OHR device and chest strap HR.

**Figure 3:**
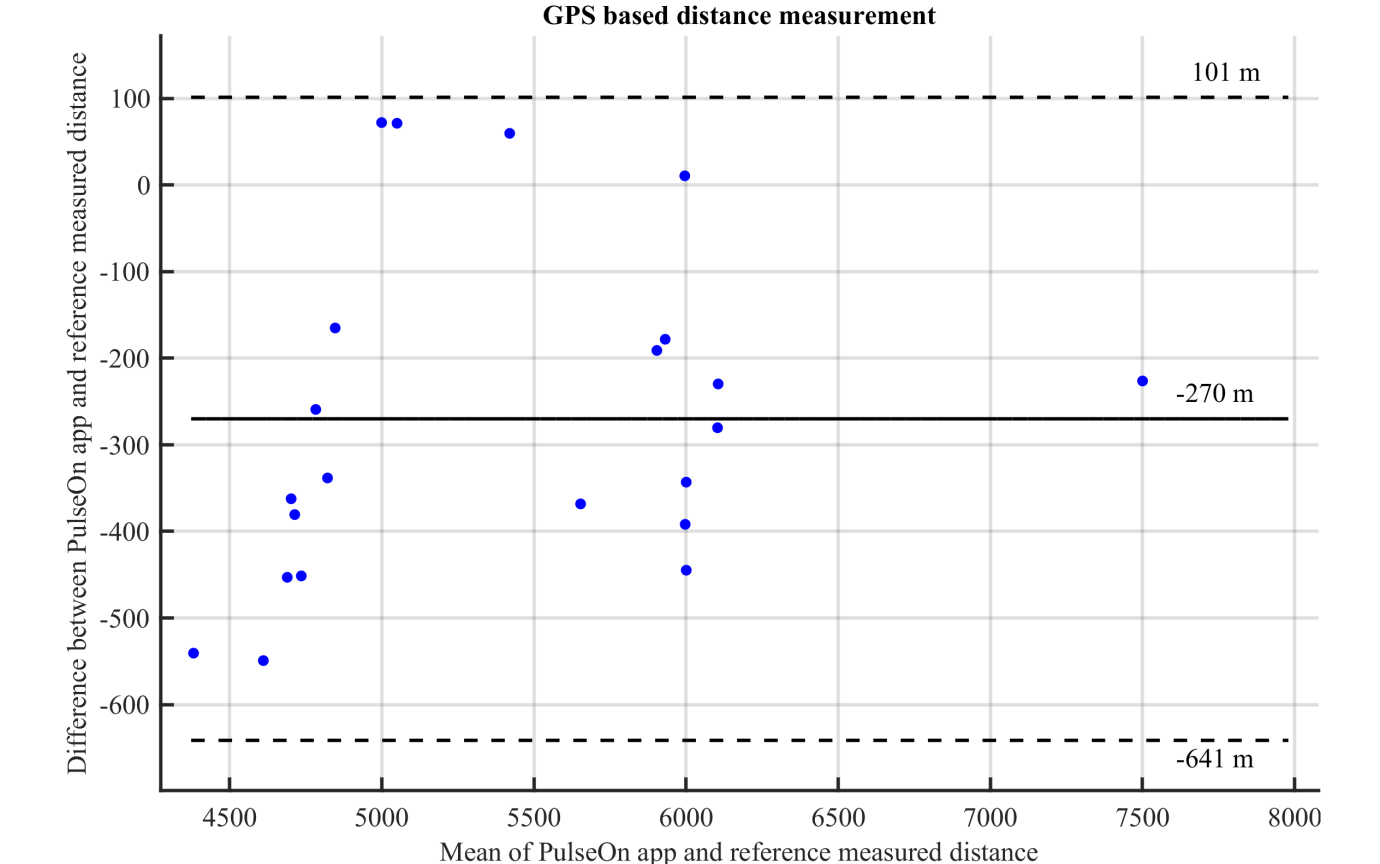
Bland−Altman plot comparing the phone GPS distance measured by the PulseOn app and a reference tracker distance estimation during outdoor running protocol (solid horizontal line: bias, dashed lines: 95% confidence limits of agreement).

### Maximal Oxygen Intake Capacity Estimation

**Figure 4:**
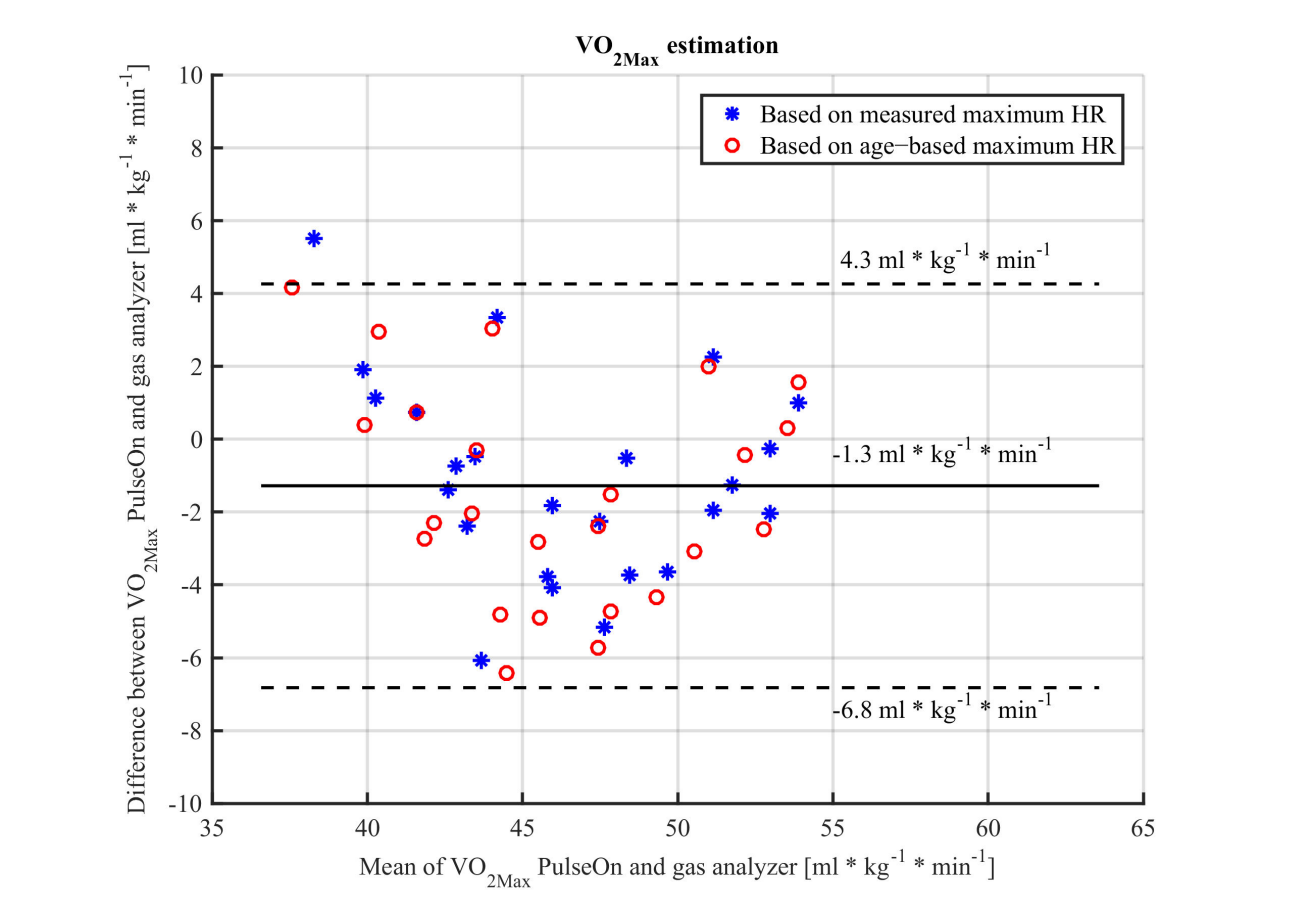
Bland−Altman plot of VO2Max estimates from the PulseOn app (wrist PPG device + phone GPS) during a submaximal exercise test versus gas analyzer based estimate during maximal exercise tests−dots represent data when age−based maximum HR is used for an estimation, while an asterix represents estimations based on true measured maximum HR (solid horizontal line: bias, dashed lines: 95% confidence limits of agreement).

VO_2Max_ estimated with a wrist PPG device and phone GPS data with a PulseOn app was close to VO_2Max_ measured during maximum exercise tests in laboratory conditions (Tables 3 and 4). VO_2Max_ estimates were slightly underestimated with the submaximal test with the PulseOn app with a MAPE of 5.2% (4.7% for males and 5.8% for females), when measured maximum HR was used in the estimation. The distance estimated by a phone GPS was underestimated on average by 5.0% (−270m) (Figure 3). However, this error did not correlate with the VO_2Max_ error. When an age-based maximum HR estimate was used, the error slightly increased (Table 4). There was no statistically significant difference between the estimates when the measured maximum HR was used in the estimation. Figure 4 presents the Bland-Altman plot of the VO_2Max_ estimates, which shows a tendency towards larger errors with lower VO_2Max_ values.

**Table 2:** Accuracy of wrist optical heart rate device during treadmill running up to maximum speed.

Activity	Reliability, %	Accuracy, %
Rest when standing	96.9	97.1
Ramp-up running	95.3	98.3
Entire protocol	95.4	98.1

**Table 3:** Maximal oxygen uptake (VO_2Max_) estimated from optical heart rate data and based on measured maximum heart rate value.

Performance metric	All (N=24)	Male (n=13)	Female (n=11)
Bias (ml ·kg^−1^·min^−1^)	−1.07	−1.28	−0.82
SD^a^ (ml ·kg^−1^ ·min^−1^)	2.75	2.42	3.19
MAE^b^ (ml ·kg^−1^·min^−1^)	2.39	2.29	2.51
MAPE^c^	5.2	4.7	5.8
Statistical test (*P* value)	.06(W^d^)	.08(T^e^)	.42(T^e^)
Correlation coefficient	ρ=0.86, (*P*<.01)(Sp^f^)	*r*=.77, (*P*<.01) (Pe^g^)	*r*=.69, (*P*<.05) (Pe^g^)

^a^SD: Standard deviation.

^b^MAE: Mean absolute error.

^c^MAPE: Mean absolute percentage error.

^d^W: Wilcoxon test.

^e^T: Paired *t* test.

^f^Sp: Spearman correlation coefficient.

^g^Pe: Pearson correlation coefficient.

**Table 4:** Maximal oxygen uptake (VO_2Max_) estimated from optical heart rate data and based on an age-based maximum heart rate estimate.

Performance metric	All (N=24)	Male (n=13)	Female (n=11)
Bias (ml ·kg^−1^ ·min^−1^)	−1.49	−1.52	−1.46
SD^a^ (ml ·kg^−1^ ·min^−1^)	2.95	2.70	3.35
MAE^b^ (ml ·kg^−1^ ·min^−1^)	2.76	2.58	2.96
MAPE^c^, %	5.9	5.2	6.8
Statistical test (*P* value)	.03(W^d^)	.07(T^e^)	.18(T^e^)
Correlation coefficient	ρ=0.87, (*P*<.01)(Sp^f^)	*r*=.73, (*P*<.01) (Pe^g^)	*r*=.63, (*P*<.05) (Pe^g^)

^a^SD: Standard deviation.

^b^MAE: Mean absolute error.

^c^MAPE: Mean absolute percentage error.

^d^W: Wilcoxon test.

^e^T: Paired *t* test.

^f^Sp: Spearman correlation coefficient.

^g^Pe: Pearson correlation coefficient.

### Energy Expenditure

**Figure 5:**
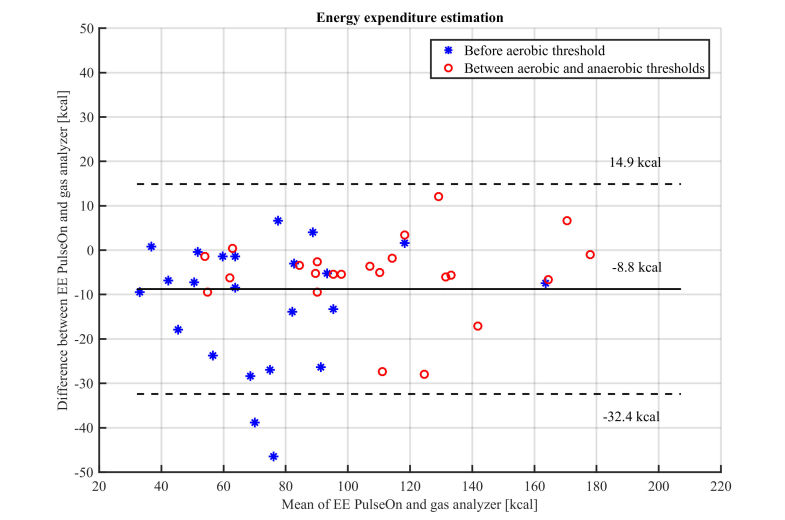
Bland−Altman plot comparing an energy expenditure estimation with a wrist PPG device and gas analyzer during a maximum exercise test−the asterisk denotes data before the aerobic threshold, while dots represent data between aerobic and anaerobic thresholds (solid horizontal line: bias, dashed lines: 95% confidence limits of agreement).

Data from one male subject was excluded from the EE estimation analysis due do failure in respiratory gas analysis data acquisition, and results are reported for the remaining 23 subjects. Error in the EE estimation was lower (MAPE 6.7%) in the higher intensity exercise (above the aerobic threshold, but below the anaerobic threshold), but increased in lower intensities (Tables 5 and 6, and Figure 5). A wrist PPG device tended to underestimate the EE during treadmill running. The correlation with respiratory gas estimated EE was high (>.93) during higher intensity exercise, especially in females.

**Table 5:** Statistical error analysis of energy expenditure during light intensity.

Performance metric	All (N=23)	Male (n=12)	Female (n=11)
Bias (kcal)	−11.93	−14.24	−9.41
SD^a^ (kcal)	13.99	16.45	10.95
MAE^b^ (kcal)	13.05	15.28	10.65
MAPE^c^, %	16.5	16.6	16.3
Statistical test (*P* value)	<.001 (W^d^)	.01 (T^e^)	.02 (T^e^)
Correlation coefficient^e^	ρ=0.77, (*P*<.01) (Sp^f^)	*r*=.88, (*P*<.01) (Pe^g^)	*r*=.79, (*P*<.01) (Pe^g^)

^a^SD: Standard deviation.

^b^MAE: Mean absolute error.

^c^MAPE: Mean absolute percentage error.

^d^W: Wilcoxon test.

^e^T: Paired *t* test.

^f^Sp: Spearman correlation coefficient.

^g^Pe: Pearson correlation coefficient.

**Table 6:** Statistical error analysis of energy expenditure during medium heavy intensity.

Performance metric	All (N=23)	Male (n=12)	Female (n=11)
Bias (kcal)	−5.58	−6.78	−4.28
SD^a^ (kcal)	9.00	12.24	3.10
MAE^b^ (kcal)	7.52	10.43	4.34
MAPE^c^, %	6.7	8.2	5.1
Statistical test (*P* value)	.007 (T^d^)	.08(T^d^)	.001(T^d^)
Correlation coefficient	*r*=.97, (*P*<.01) (Pe^e^)	*r*=.93, (*P*<.01) (Pe^e^)	*r*=.99, (*P*<.01) (Pe^e^)

^a^SD: Standard deviation.

^b^MAE: Mean absolute error.

^c^MAPE: Mean absolute percentage error.

^d^T: Paired *t* test.

^e^Pe: Pearson correlation coefficient.

## Discussion

### Principal Findings

We estimated HR, EE, and VO_2Max_ based on wrist PPG and phone GPS speed and evaluated their accuracy during running based on golden reference methods. OHR appeared to be accurate during running; the MAPE was 1.9% and reliability 95.4% during a maximal voluntary exercise test. This is well in line with the earlier results [4] and suggests that high-end consumer-grade OHR devices are capable of accurately monitoring HR during running, even up to a maximum HR.

The accuracy of more advanced parameters estimated from OHR is dependent, both on the accuracy of the OHR and on the validity of the analytical models. We used an HR-based estimation of the EE, and an HR and running speed-based estimation of the VO_2Max_ developed earlier by a company (Firstbeat, Jyväskylä, Finland), which is widely available in various sports products. EE estimation with this method has been validated earlier [8], suggesting a slight underestimation of EE by 13% when a chest strap HR was used. In our study, the overall EE estimation accuracy is well in line with this. EE estimation was the most accurate during medium or hard intensity with a MAPE of 6.7% (males 8.2% and females 5.1%). During light intensity, the error increased to 16.5% (males 16.6% and females 16.3%). Differences in the EE estimation based on HR may be related to individual differences in the basic metabolism, a thermogenesis effect due to diet or metabolic effect, which affects the body mass ratio [25]. For comparison, 10.1-18.2% MAE has been reported for EE estimation by activity trackers [26]. The EE estimates based on OHR and indirect calorimetry had strong correlations for all (N=23) subjects during light intensity (ρ=0.77), while at a higher intensity their correlation was close to 1 (*r*=.97). The results are comparable to a similar study by Robertson et al [12], who used a chest strap HR with the same EE estimation method [18] and reported moderate (*r*=.57) to strong (*r*=.85) correlations during low and high intensity exercise, respectively. However, significant differences between different OHR devices have been reported. Recently, Wallen et al studied the EE estimation accuracy of four different OHR devices against indirect calorimetry and found only one device (Samsung Gear S) to have a strong correlation (*r*=.86) with the reference, while the other three devices exhibited only a weak correlation to reference EE [13]. Our results suggest that a wrist-worn OHR may offer a similar estimation of true EE during running to chest strap HR based methods when a high quality OHR device and proper physiological model are applied in EE estimation.

The level of fitness may be quantified by the estimation of VO_2Max_. We used OHR and a mobile-based speed estimation to estimate VO_2Max_ during self-paced outdoor running in real and challenging outdoor conditions during winter in Finland. These conditions may be considered the “worst case” training conditions and, for example, the temperature difference may increase the observed estimation error for VO_2Max_. The analytical method was based on the well-known HR versus speed relationship and on detecting the most reliable data periods for VO_2Max_ estimation during the exercise [20]. We compared this estimate with the golden standard of the VO_2Max_ estimation, that is, respiratory gas analysis acquired during a maximal voluntary exercise test in a sports laboratory. The results suggest that OHR and speed-based VO_2Max_ estimation during self-paced running are able to quite accurately estimate VO_2Max_, even in these challenging outdoor conditions; we found a MAPE of 5.2% (males 4.7% and females 5.8%) for VO_2Max_ when an individually measured HR maximum was used in the estimation. When age-estimated maximum HR was used, the error increased slightly. A significant contribution to the inaccuracy originated from phone GPS tracking, which underestimated the distance by 5% on average and led to a corresponding underestimation of the VO_2Max_. In addition, during the outdoor testing, there were challenging weather conditions (cold and winter), which posed challenges for PPG HR estimation because of potentially poor perfusion, increasing the potential error for the OHR during field conditions. These weather conditions may also have affected the real VO_2Max_. Also, differences in running efficiency affect the correspondence between the running speed and the true physical load, and, hence, increase the error in HR and speed-based VO_2Max_ estimation. There was also a tendency for the OHR and speed-based analysis to overestimate the VO_2Max_ in individuals with a lower real VO_2Max_. In summary, the results suggest that the method may be used to estimate VO_2Max_ relatively accurately during self-paced running, even in challenging outdoor conditions.

### Limitations and Strengths

This study has several strengths, but also weaknesses. To our knowledge, this is the first study to report both EE and VO_2Max_ estimation accuracy, based on OHR data. We used a realistic or even challenging setting (self-paced outdoor running in winter) to estimate VO_2Max_. This is a setting that can be applied by an ordinary user, and as such, the method can be directly applied by healthy users to estimate their fitness levels. We used the golden standard (gas analyzer and controlled sports laboratory with maximal voluntary exercise) as a reference for EE and VO_2Max_. The main weakness of the study is that it had a relatively small study population; however, despite this, the results can be considered to be at least indicative. In addition, the outdoor tests were carried out in a challenging environment (winter, cold, and sometimes potentially slightly slippery roads), increasing the error of the outdoor VO_2Max_ estimation. On the other hand, this provides the worst case scenario, and the results were still within an acceptable error margin. Finally, the study included only one wrist OHR device, which limits the generalizability of the results. Only a single device was used for practical reasons—wearing several devices in both laboratory and outdoor conditions would have complicated the study implementation. The PulseOn device was chosen for the study because, at the time of data collection, to our knowledge, other available wrist OHR devices did not support estimation of VO_2Max_ together with accurate data logging capability. However, the results are not without generalizability. The applied VO_2Max_ and EE estimation algorithm [18,20] has been validated with a chest strap HR monitor [12], is commercially widely available, and could be applied with other accurate OHR devices as well. Hence, we do not consider the results of the study to be specific to applied wrist devices only, but to OHR technology in general.

### Conclusions

We applied a commercially available OHR device to estimate HR, EE, and VO_2Max_ during running and evaluated their accuracy against golden standard methods. The results show that current high-end wrist OHR devices may provide accurate HR that can be compared with a chest strap HR, during running, up to a maximum HR. When combined with proper analytics, OHR may be used to quite accurately estimate EE, especially during moderate to medium heavy intensity activities. An estimation of VO_2Max_ during self-paced outdoor running using OHR and a mobile phone’s GPS data and proper HR analytics also allows a relatively accurate estimation of a fitness level (VO_2Max_). Wrist PPG devices accompanied by phone apps provide a reliable alternative for training monitoring in realistic conditions.

## Abbreviations

HR: Heart rate
PPG: Photoplethysmography
OHR: Optical heart rate
BPM: Beats per minute
MAE: Mean absolute error
MAPE: Mean absolute percentage error
SD: Standard deviation
EE: Energy expenditure
VO2Max: maximal oxygen consumption
